# Discrimination of Exudative Pleural Effusions Based on Pleural Adenosine Deaminase (ADA)-C-Reactive Protein (CRP) Levels, and Their Combination: An Observational Prospective Study

**DOI:** 10.3390/jpm11090864

**Published:** 2021-08-30

**Authors:** Garifallia Perlepe, Charalampos Varsamas, Efthymia Petinaki, Dionysios Antonopoulos, Zoe Daniil, Konstantinos I. Gourgoulianis

**Affiliations:** 1 Department of Respiratory Medicine, Faculty of Medicine, University of Thessaly, 41110 Larissa, Greece; harrisvarsamas@gmail.com (C.V.); zdaniil@uth.gr (Z.D.); kgourg@med.uth.gr (K.I.G.); 2 Department of Microbiology, University Hospital of Larissa, 41110 Larissa, Greece; petinaki@med.uth.gr; 3 Department of Biochemistry and Biotechnology, School of Health Sciences, University of Thessaly, 41110 Larissa, Greece; diadonop@uth.gr

**Keywords:** pleural effusions, malignant pleural effusions, parapneumonic pleural effusions, tuberculous pleural effusions, pleural biomarker, lung ultrasound, pleural CRP, pleural ADA

## Abstract

(1) Background: Malignant (MPE), parapneumonic (PPE) and tuberculous (TPE) pleural effusions constitute common causes of pleurisy. Discriminating among them is usually challenging. C-reactive protein (CRP) and adenosine deaminase (ADA) pleural levels (p-CRP, p-ADA) have been used as differentiators in many studies showing promising results. This study aims to evaluate the diagnostic value of p-CRP, p-ADA levels and their combination among the three categories. (2) Methods: A prospective study of 100 patients with MPE (*n* = 59), PPE (*n* = 34) and TPE (*n* = 7) from a single centre was performed. p-CRP levels were evaluated between PPE and non-PPE and between complicated (CPPE) and non-complicated PPE. ADA levels were also measured to classify patients among MPE and non- MPE. Eventually, the combination of p-CRP and p-ADA values was used as a discrimination factor among PPE, MPE and TPE. (3) Results: ROC analysis revealed that p-CRP with a cut-off value: 4.4 mg/dL can successfully differentiate PPE (AUC = 0.998). The cut-off level of 10 mg/dL can predict CPPE with sensitivity: 63%, specificity: 71.4%, positive predictive value (PPV): 89%, and negative predictive value (NPV): 33%. Furthermore, patients with ADA levels ≤ 32 U/L were more likely to belong to the malignant group sensitivity: 93%, specificity: 78%, PPV: 85.9%, and NPV: 88.9%. Discriminant analysis showed that the combination of p-CRP and p-ADA levels can discriminate PPE, MPE and TPE in 93% of cases. (4) Conclusion: This study provides evidence that p-CRP and p-ADA levels could be possibly used in clinal practice in order to establish a diagnosis among MPE, PPE and TPE.

## 1. Introduction

It is estimated that pleural effusion develops in more than 1.5 million patients each year in the United States, with a wide variety of potential causes [1]. The incidence is increasing parallel with the need of systematic evaluation, as the treatment and prognosis of pleural effusions largely depend on the cause. A delay in the diagnosis and initiation of proper treatment leads to increase in the complication rate, mortality and morbidity, especially concerning parapneumonic effusions [2]. Establishing the underlying cause is not always achievable using the conventional methods, so alternative tests are needed. Several pleural fluid biomarkers have been used towards this direction. The ideal biomarker should be easily measured, at a reasonable cost, and should aid in decision making [3]. 

C-reactive protein (CRP) known as “acute-phase protein,” is synthesized by the liver in response to various stimuli and produced early in the inflammatory process. CRP is increased in the serum/plasma of patients with pneumonia and many studies also confirmed that increased pleural CRP (p-CRP) may present a possible biomarker for pleural infection, although with inconclusive results [4,5,6]. Additionally, high CRP levels (cut-off: 10 mg/dL) have been used as differentiator between complicated and non-complicated parapneumonic pleural effusions, with comparable AUC scores to those of widely accepted pleural pH and glucose [7]. 

The diagnosis of tuberculous pleural effusion (TPE) is usually difficult as simple thoracentesis rarely leads to the isolation of *Mycobacterium tuberculosis* (<10%) [8]. Pleural fluid adenosine deaminase (ADA) is probably the simplest, most widely used and repeatable among the various biomarkers evaluated for diagnosing TPE [9]. A meta-analysis of 63 studies including 2796 patients with tuberculous pleuritis reported that the sensitivity and specificity of ADA in the diagnosis of pleural TB were 92% and 90%, respectively [10].However, ADA levels > 40 IU/L can indicate empyema, lymphomas or collagen vascular diseases, adding complexity to the final diagnosis [11]. Interestingly, ADA levels among malignant pleural effusions (MPE) are extremely low and rarely exceed 40 IU/L, adding a potential tool for discrimination among tuberculous and malignant pleural effusions. 

The aim of this study was to determine whether the pleural levels of two biological markers, ADA- CRP, and their combined function, could help us discriminate malignant (MPE), parapneumonic (PPE) and tuberculous (TPE) pleural effusions. Malignant disease involving the pleura and parapneumonic effusion are the leading causes of exudative pleural effusions. However, the diagnosis of tuberculous pleuritis should also be considered in any patient with an exudative pleural effusion. Recommended best current practise of distinguishing among the three categories is not established, as a combination of biomarkers, cytology, laboratory tests, estimation of response to treatment, and clinical judgement are necessary to reach a diagnosis. In occasion of diagnostic uncertainty, invasive methods such as medical thoracoscopy or VATS are required, if the patient’s performance status allows it. ADA constitutes an important, easily measured pleural fluid biomarker with a considerable diagnostic yield in diagnosing tuberculosis in several human fluids including pleural fluid [12]. Additionally, CRP is an acute phase marker that exhibits elevated expression during inflammatory conditions such as rheumatoid arthritis, some cardiovascular diseases, and infection. It is widely measured in plasma samples in everyday clinical practice and easily available, and its levels can be elevated in sites of inflammation, such as pleura [13]. To our knowledge, the synergic action of ADA and pleural CRP has been mentioned in one previous study. ADA and CRP concentration levels were found to be the most important parameters for discrimination among the three categories when compared to several pleural biomarkers [14].

## 2. Materials and Methods

### 2.1. Study Population 

**Figure 1:**
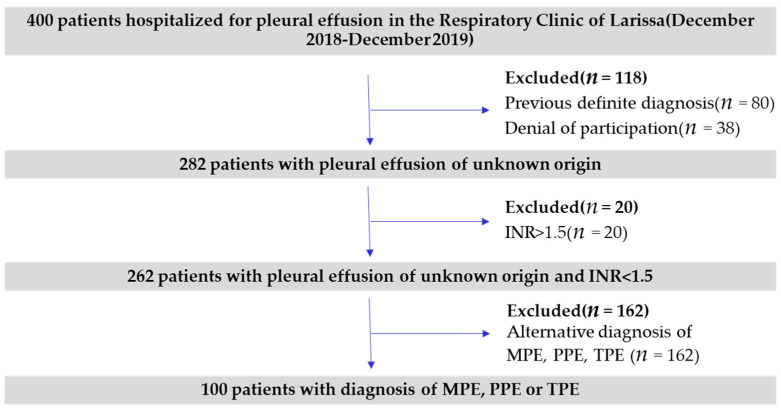
Flowchart of patients participating in the study. INR = international normalized ratio, MPE: malignant pleural effusion, PPE = parapneumonic pleural effusion, TPE: tuberculous pleural effusion.

The present study included 100 patients, admitted to the department of Respiratory Medicine at University Hospital of Larisa, Greece, between December 2018 and December 2019. All the participants received a diagnosis of MPE, PPE or TPE during hospitalization or at post hospitalization surveillance. The exclusion criteria for this study were a previous definite diagnosis before investigation, coagulopathy (defined by INR > 1.5) or both (Figure 1). Non-urgent pleural aspirations and chest drain insertions should be avoided in anticoagulated patients until (INR) <1.5 [15].

The diagnosis of malignant effusion was established by the presence of malignant cells on pleural fluid cytological examination or in a biopsy specimen acquired during thoracoscopy taking place in our department. The pleural effusion was considered parapneumonic when it was associated with pulmonary infiltrates responsive to antibiotic treatment, fever, purulent sputum or when a microorganism was identified in the pleural fluid. Among parapneumonic effusions, CPPE (complicated parapneumonic pleural effusions) were certified when pleural fluid was obviously pus(empyema),and when pH was lower than 7.2 and/or glucose was lower than 40mg/dl [16]. TPE was diagnosed based on positive pleural fluid or pleura tissue cultures for mycobacterium tuberculosis or when pleural effusion was co-existing with positive cultures for mycobacterium tuberculosis in samples by other sites or when the pleural biopsy specimen revealed typical epithelioid cell.

All patients gave informed consent and the study was approved by the ethics committee of the Institute of Clinical Research (University Hospital of Larissa, Larissa, Greece). The participants of this study had a scheduled and appropriate follow up to ensure response to treatment or recovery. 

A diagnostic or palliative thoracentesis was made based on the patient’s symptoms using a lung ultrasound to avoid any complications. Each pleural effusion was characterized based on the ultrasound image as anechoic, complex non-septate, complex septate, homogeneously hyperechogenic. [17]. The effusion size was determined as small (occupying less than one-third of the visualized hemithorax), moderate (occupying one-third to two-thirds of the hemithorax), or large (occupying more than two-thirds of the hemithorax) as illustrated in chest CT [18]. The sample used for analysis was collected from the first thoracentesis, before the use of antibiotics.

The primary outcomes of this study were: (1) The capacity of both ADA and CRP pleural levels to discriminate among the three categories of pleural effusions included in this study: parapneumonic pleural effusions (PPE), malignant pleural effusions (MPE) and tuberculous pleural effusions. (2) The ability p- CRP levels to differentiate between PPE and non-PPE.

Secondary outcomes constitute: (1) ADA levels as a differentiator among MPE and non-MPE (2) CRP levels with a cut of: 10 mg/dL as a discriminative factor among non-complicated and complicated pleural effusions. 

### 2.2. Laboratory Tests

Pleural samples were analysed for total differential cell counts, CRP, ADA, glucose, total protein, albumin, lactate dehydrogenase (LDH), pH. For all pleural specimens cytologic examination and bacterial cultures were obtained. Furthermore, samples were examined for mycobacteria using Ziehl-Neelsen stain and culture. 

The supernatant of each sample was obtained by centrifugation at 300 rpm for 15 min and stored at 20 °C until being assayed for the CRP measurement. CRP measurements were performed by particle-enhanced immunoturbidimetric assay with the cobas c 702 analyzer, using the Tina-quant C-Reactive protein IV kit (Roche Diagnostics, Mannheim, Germany). The appropriate control was provided by the same company and assays were performed according to the manufacturer’s instructions by the Department of Microbiology, University Hospital of Larissa, Larissa, Greece. The measuring range was 0.6–350mg/dl. 

Total pleural fluid ADA was determined by the Giusti method which is based on the measurement of ammonia released from adenosine when converted to inosine. Pleural fluid was centrifuged and the supernatant was incubated in adenosine buffer at 37 °C, followed by incubation with Berthelot reagent at 37 °C and subsequent photometric analysis at 405 nm using a Secomam Basic semi- automatic analyser. 

### 2.3. Statistical Analysis

For the descriptive statistical analysis, the continuous variables were expressed as mean (with standard deviation) and median (with the 25th and 75th percentiles), while the discrete variables as frequency (relative percentage frequency). The accuracy of pleural fluid biochemistries in distinguishing between groups was established by calculating sensitivity, specificity, and likelihood ratios (LR). The ROC (Receiver Operating Characteristic) analysis was used to investigate a discrimination threshold. Youden’s index was used to find the optimal cut-off point. The chi-square test was used to make comparisons between nominal and ordinal variables. Discriminant analysis was used to classify pleural effusions based on ADA and CRP levels. Wilks’ Lambda value was set to indicate greater discriminatory ability. Normal distribution was examined by the Shapiro-Wilk test. 

Data processing was performed using statistical analysis program IBM SPSS version 25. A *p*-value less than 0.05 was considered statistically significant.

## 3. Results

### 3.1. Patients Characteristics 

The study sample consists of 100 patients (28 women and 72 men). The median age was calculated to be 68 years (Q25 = 57.5, Q75 = 75). Mean values for CRP and ADA were 2.8 mg/dL (Q25 = 1.3, Q75 = 7.6) and 21.8 U/L (Q25 = 12, Q75 = 45), respectively. Among participants 59% have been diagnosed with MPE, 34% with PPE and 7% with TPE. CPPE constitutes a subgroup of PPE corresponding to 79.4 of total. Regarding the size of pleural effusion based on radiological image: 44% were classified as large, 33% as median, and 23% as small. Large effusions were more common among the PME, without statistical significance (*p* = 0.068). The ultrasound staging revealed 47 anechoic effusions, 41 complex non-septate, 26 complex septate and 6 homogeneously hyperechogenic. MPE presented more frequently as anechoic effusions, UPPE as complex non-septate and CPPE as complex-septate. TPE did not present with a special ultrasound image. Medical thoracoscopy was performed to establish a diagnosis in 14% of cases. Table 1 summarizes the characteristics of the sample.

### 3.2. Discriminating PPE Based on p-CRP Levels

**Figure 2:**
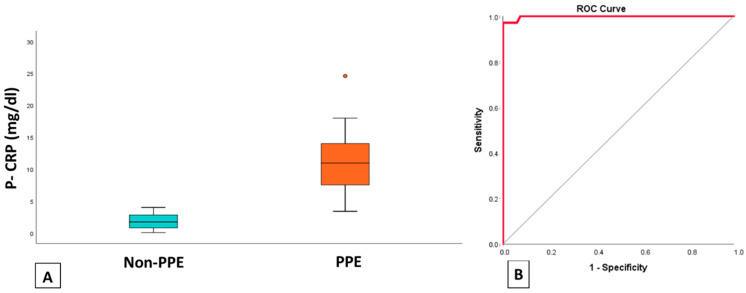
(**A**) Boxplot of p-CRP levels of PPE and non-PPE. (**B**) ROC curve of CRP levels for differentiating parapneumonic pleural effusions from other types of pleural effusions. PPE: Parapneumonic pleural effusions, p-CRP: pleural C-Reactive protein.

The mean value of p-CRP for patients with PPE was 10.91 mg/dL and for patients with alternative diagnosis 1.77 mg/dL. We observe, therefore, that patients receiving a diagnosis of PPE have significantly higher p-CRP levels than patients with another diagnosis of pleural effusion (Figure 2). CRP levels had a high diagnostic accuracy for identifying parapneumonic effusions as measured by the area under ROC curve (AUC = 0.998) (Figure 2). The pleural fluid CRP cut-off value for differentiating among parapneumonic effusions and the other groups was 4.4 mg/dL. The sensitivity, specificity, positive predictive value, and negative predictive value were 97%, 67%, 100%, and 98.5%, respectively.

**Figure 3:**
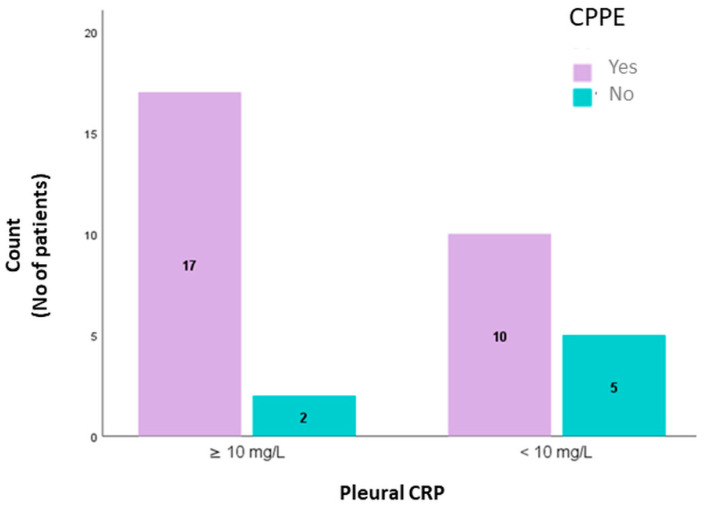
Distribution of patients in CPPE and non- CPPE groups based on a cut-off of pleural CRP ≥ 10 mg/dl. CRP: C-reactive protein, CPPE: complicated parapneumonic pleural effusion.

Estimating the cut-off level of 10 mg/dL we saw that it can act as a differentiator between uncomplicated parapneumonic effusions (UPPE) and CPPE with sensitivity: 63%, specificity: 71.4%, positive predictive value:89%, and negative predictive value:33%. p-CRP levels in two groups are seen in Figure 3.

### 3.3. ADA Levels among MPE and Non-MPE

**Figure 4:**
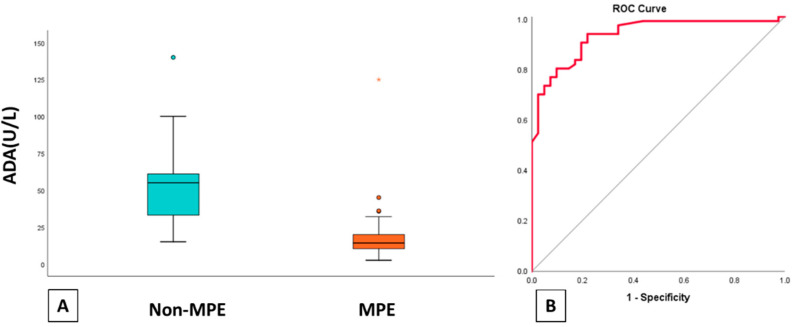
**A**. Boxplot of ADA levels of non-MPE and MPE. **B.** ROC curve of ADA levels for differentiating malignant pleural effusions from other types of pleural effusions. MPE: malignant pleural effusions, ADA: adenosine deaminase.

The mean value of the ADA-pleural for patients with a diagnosis of malignant pleurisy is 18.11 U/L, while for patients with another diagnosis of pleural effusion 53.58 U/L. We observe, therefore, that patients diagnosed with malignant pleural effusion have significantly lower ADA values than patients diagnosed with PPE and TPE. The box plot of Figure 4 illustrates that ADA values for patients diagnosed with malignant pleurisy are more concentrated and lower. ROC analysis showed that ADA can successfully discriminate MPE from the other two categories and AUC was 0.927. Cut off-point was measured 32.5. Ιn conclusion ADA levels ≤ 32.5 U/L can discriminate MPE with 93% sensitivity, 78% specificity, 85.9% positive predictive value, and 88.9% negative predictive value.

### 3.4. ADA and CRP Levels as Differentiators between PPE. MPE, TPE

Initially, since there are three diagnostic categories (PPE, TPE, and MPE) in the dependent variable, we created two functions:

F1 = β01 + β11 × CRP + β21 × ADA(1)

F2 = β02 + β12 × CRP + β22 × ADA(2)

Function 1 is related to CRP and Function 2 is related to ADA. Continuously, it is useful to create a diagram between the two variables. We noticed that patients with pleural effusion, low CRP (mean = 1.7), and low ADA (mean = 18.1) tend to be classified as MPE. Patients with pleural effusion, low CRP (mean = 2.3), and moderate-high ADA values (mean = 74.3) tend to be classified as TPE. Patients with pleural effusion, mean-high CRP values (mean = 10.9), and moderate-high ADA values (mean = 49.3) tend to be classified in the PPE category. The result of the discriminant analysis shows that for the two variables (CRP and ADA) the mean values in the three diagnostic categories (PPE, TPE, MPE) differ statistically significantly. In addition, the Wilks lambda index informs us about the differences in the diagnostic categories. Thus, the *p*-value for both markers (CRP and ADA), is less than 5%. This suggests that both variables are important prognostic factors. Based on the results of the table of unstandardized canonical discriminant function coefficients, the two functions (3), (4) have been transformed as follows:

F1 = −2154 + 0.333 × CRP + (−0.152) × ADA(3)

F2 = −0.767 + 0.016 × CRP + 0.046 × ADA(4)

**Figure 5:**
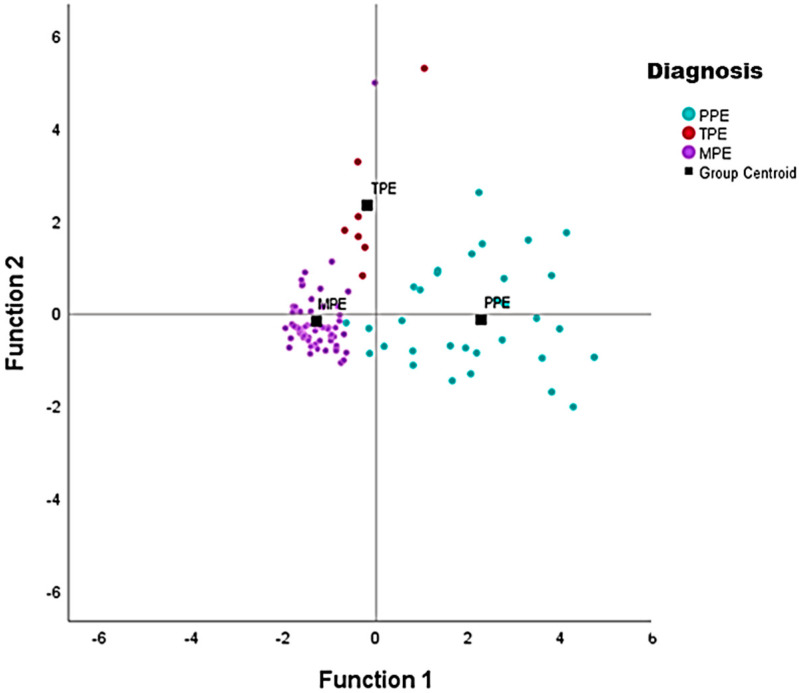
Group centroid diagram of mean values of ADA and CRP for PPE, MPE and TPE. ADA: adenosine deaminase, CRP: C–reactive protein, PPE: parapneumonic pleural effusions, MPE: malignant pleural effusions, TPE: tuberculous pleural effusions.

Based on the Standardized Canonical Discriminant Function Coefficients, both indicators are equally important in terms of their contribution to the separation function. Finally, the group centroid table (Table 1) gives us the mean value of each divisor function for each group: PPE (2.278, −0.147), TPE (−0.190, 2.320), and MPE (−1.290, −0,190). The percentage of successful discrimination was calculated to be 93% (Figure 5).

## 4. Discussion

The present study provides evidence for the utility of the pleural fluid CRP and ADA measurements in discriminating among PPE, MPE and TPE. The latter remain the most frequent causes of exudative pleural effusions [19]. Several biochemistries have been widely used in differentiating between the three categories. Pleural pH levels are lower among empyemas and parapneumonic effusions, although low pH levels could also be observed in TPE and MPE [20]. Pleural fluid for total white blood cell count and differential cell count heightens suspicion for PPE in case of a neutrophilic predominance. In contrast, MPE should be considered even in patients with neutrophilic exudative effusion [21]. Moreover, glucose pleural levels <60 mg/dL could be typical for both PPE and TPE [22]. Thus, the difficulty in discriminating among the three categories is indisputable.

CRP is a widely recognised inflammation marker and several studies have shown elevated serum levels in patients with parapneumonic effusions when compared to healthy individuals, other exudates and transudates [23].p-CRP levels have shown promising results in discerning PPE. In this study we found that p-CRP levels with a cut off of 4.4 mg/dL can exceptionally distinguish PPE and AUC, sensitivity, specificity, positive predictive value, and negative predictive value were 0.998, 97%, 67%, 100%, and 98.5%, respectively. 

J Porcel et al., showed that p-CRP had a high diagnostic accuracy for PPE (AUC= 0.82), and when accompanied by neutrophilic predominance, CRP levels ≥ 4.5 mg/dL could almost always predict a PPE [7]. Two other studies from our department have also shown significantly discriminative properties for p-CRP in the past [14,24]. S. Izhakian et al., set a cut off of 1.38 mg/dL for parapneumonic effusions that yielded 84.2% sensitivity, 71.5% specificity, 37.6% positive predicted value, and 95.6% negative predicted value. Finally, a meta-analysis that assessed the diagnostic performance of pleural CRP for PPE showed sensitivity: 80% specificity 82%. The AUC was measured to be 0.88, although the authors highlight the high heterogeneity among included studies [5]. 

In our study we also assessed the accuracy of CRP for CPPE. The results showed that CPPE had p-CRP levels > 10 mg/dL more often than UPPE and the diagnostic performance of CRP was comparable to a previous meta-analysis by Dajiang Li et al. with the same cut off [5]. PPV of 89% indicates that CRP levels ≥ 10 mg/dL could be an indicator for drainage as only 11% of patients would not benefit. The lack of high sensitivity level, on the other hand, is mentioned also in the study of Porcel et al., suggesting that pleural CRP > 10 mg/dL, in combination with either pH < 7.20 or glucose < 60 mg/dL would ameliorate the statistical parameters [7]. 

We also hypothesized that low ADA levels could discriminate MPE from the other categories as the non- malignant group was composed of UPPE, CPPE and TPE that are characterized by high ADA levels. Patients with ADA levels < 32.5 U/L in our study group were more likely to belong to the malignant group. Therefore, we underline another utility of ADA besides its known role in tuberculous pleurisy. Nevertheless, we have to underline that lymphomas were not included in the malignant group, as numerous studies have demonstrated high p-ADA levels among lymphoma pleural effusions as a response to lymphocytes proliferation [25].

Finally, we attempted to categorize pleural effusions based on both ADA and CRP levels. The statistical analysis revealed outstanding discriminating ability that accedes to 93%. Mean values for CRP levels and ADA levels were measured as: {1.7 mg/dL–18.1 U/L}, {2.3 mg/dL–74.3 U/L}, {10.9 mg/dL–49.3 U/L}, for MPE, TPE and PPE respectively. To summarize, low CRP and low ADA levels are characteristic of MPE, low CRP and moderate-high ADA values raise suspicion of TPE and high CRP and moderate-high ADA values render the diagnosis of PPE among the three categories of this study, Daniil et al. [14], were the first to identify that the combinatorial effect of ADA and CRP was beneficial as differentiator among the three categories. In their study, among seven biomarkers that have been investigated, CRP and ADA had the best results. More precisely, an individual with an ADA concentration level of >45 U/L and a CRP concentration < 4 mg/dL was more likely to belong to the TPE group, whereas one with an ADA concentration level < 40 U/L and a CRP concentration > 6 g/dL was more likely to belong to the PPE group, and one with a CRP concentration < 4 mg/dL to the MPE group. Comparing the results of the two studies, we reaffirmed the hypothesis that ADA and CRP pleural levels could categorize PPE, MPE and TPE and the outcomes were comparable, with exception of higher ADA levels in the PPE group. The difference is probably explained by the high percentage of CPPE among PPE in our study group, that is related to higher ADA levels. 

The main limitation of the present study is the respectively small size sample of TPE and UPPE as well as the exclusion of lymphomatous effusions. Definitely, further studies and RCT’s are required to verify our results and identify the best combination markers for analysis. The use of biomarkers in pleural effusions has been proposed as an alternative noninvasive mean of establishing a diagnosis of pleural effusion, avoiding surgical procedures in many studies. However, their use in clinical practice remains controversial. The main strength of this study is the possibility of diagnosis and discrimination of pleural effusions using cost-effective and widely available biomarkers (p-ADA, p-CRP). We suggest that p-CRP, p-ADA measurements and their combination could be easily applicable in everyday clinical practice and could offer diagnostically useful information. 

## Figures and Tables

**Table 1:** Patient Characteristics.

		Total Number	Percentage (%)
Gender	Female	28	28.0
	Male	72	72.0
Diagnosis	PPE	34	34.0
	TPE	7	7.0
	MPE	59	59.0
Pleural effusion size (Chest CT, portion of visualized hemithorax)	<1/3	23	23.0
	1/3–2/3	33	33.0
	>2/3	44	44.0
Ultrasound Image	Anechoic	47	47.0
	Complex non-septate	21	21.0
	Complex septate	26	26.0
	Homogeneously Hyperechogenic	6	6.0

## Data Availability

All data are available after request.
